# Optimal timing of surgery in head and neck squamous cell carcinoma after neoadjuvant immunochemotherapy

**DOI:** 10.3389/fonc.2026.1742883

**Published:** 2026-02-06

**Authors:** Hanbo Seng, Yunli Fan, Rui Zhao, Yanyan Liu, Jinping Meng, Ziqi Wang, Yanyan Chen, Shengli Shao, Dongjie Seng

**Affiliations:** 1 Department of Otolaryngology Head and Neck surgery, The First Affiliated Hospital of Zhengzhou University, Zhengzhou, China; 2 Department of Head Neck and Thyroid, The Affiliated Cancer Hospital of Zhengzhou University & Henan Cancer Hospital, Zhengzhou, China; 3 Department of Otolaryngology Head and Neck surgery, Henan Children’s Hospital, Zhengzhou Children’s Hospital, Zhengzhou, China

**Keywords:** disease free survival, head and neck squamous cell carcinoma, neoadjuvant immunochemotherapy, pathologic response, surgical complication, time to surgery

## Abstract

**Objectives:**

Our goal was to evaluate the influence of the interlude between neoadjuvant immunochemotherapy (NAIC) and surgery on both pathologic responses and surgical outcomes in head and neck squamous cell carcinoma (HNSCC).

**Methods:**

Patients undergoing surgery for HNSCC following NAIC were retrospectively enrolled and determined based on the time to surgery (TTS). Impact of TTS on major pathologic response (mPR), pathologic complete response (pCR), surgical complication and 3-year disease free survival (DFS) was evaluated.

**Results:**

A total of 356 patients were enrolled. 225 patients (63.2%) achieved a mPR, among whom a pCR was achieved in 104 patients (29.2%). When compared to the TTS<1 week group, patients with a TTS of 1-2 weeks and those with a TTS of 2-3 weeks exhibited comparable rates of pCR and mPR achievement; however, patients who underwent surgery more than 3 weeks after the completion of NAIC had a significantly reduced likelihood of achieving total tumor regression by 16% (95% CI: 1%-23%) and lower probability of major tumor regression by 21% (95% CI: 5%-36%). The TTS >3 weeks group bore an additional 0.67-fold risk of experiencing surgical complications and 1.23-fold increased risk of adverse recurrence or death events compared to TTS <1 cohort.

**Conclusion:**

A TTS exceeding 3 weeks was independently associated with a diminished likelihood of achieving both pCR and mPR, an increased rate of surgical complications, and a shorter duration of DFS. These findings suggest that the interval between surgery and the completion of NAIC may be optimal when kept within 3 weeks in HNSCC, though this warrants prospective validation.

## Introduction

Head and neck squamous cell carcinoma (HNSCC) ranks as the sixth most prevalent malignant neoplasm among all solid tumors (1). Over half of the cases present with lymph node metastasis at the time of initial diagnosis, and the therapeutic approach typically involves thorough resection, complemented by adjuvant treatment (2). Despite the administration of surgery-centric systemic therapy, a considerable number of patients continue to experience treatment failure, with the five-year overall survival rate lingering between 40% and 60% (3), necessitating the development of more efficacious interventions.

Given the substantial survival advantage offered by immunotherapy in recurrent or metastatic HNSCC (4), considerable attention has turned toward its role in neoadjuvant therapy. A series of clinical trials (5–8) have reported promising outcomes, including organ preservation, over 60% major pathologic response (mPR), and approximately 30% pathologic complete response (pCR). Nevertheless, much remains to be elucidated concerning the factors that influence these pathologic responses. Local modifications within tumor lesions and systemic alterations are influenced by the body’s inflammatory response and the post-neoadjuvant therapy physical condition of patients, which in turn can impact surgical outcomes. It is imperative to recognize that these factors are subject to change over the course of time. Consequently, the determination of the optimal timing for surgery is crucial in enhancing surgical efficacy. The timing of surgery subsequent to neoadjuvant immunochemotherapy (NAIC) in HNSCC has not been extensively explored in the literature. However, within the realm of other oncological entities, there is a variance in opinion regarding the correlation between an extended interval from NAIC to surgery and the incidence of a higher pCR rate, as opposed to an improved prognostic outcome. Some experts advocate that a prolonged interval between NAIC and surgical intervention is associated with an elevated pCR rate, yet this is not necessarily indicative of enhanced survival rates (9). Conversely, others contend that an extended interval does not augment the pCR rate and may even portend a less favorable prognosis (10).

Thus, the objective of our investigation was to evaluate the influence of the interlude between NAIC and surgery on both pathologic responses and surgical outcomes, with the intent of exploring the association between the timing of surgical intervention and clinical outcomes, which may help inform the optimal scheduling of surgery.

## Patients and methods

### Ethical approval

This study was approved by Our Hospital Institutional Research Committee, and written informed consent for medical research was obtained from all patients before starting the treatment. All methods were performed in accordance with the relevant guidelines and regulations.

### Investigation protocol

To address our purpose, a retrospective analysis of patients receiving curative surgery for primary HNSCC after NAIC between October 2019 and December 2024 in a tertiary cancer center was performed. Enrolled patients must underwent at least two cycles of NAIC, but those with a prior history of cancer were excluded. Then data regarding demography, pathology, treatment, and follow-up was collected.

### Variable definition

Tumor and nodal staging were determined in accordance with the 8th edition of the AJCC staging system. Pathological differentiation was classified into well-differentiated, moderately differentiated, or poorly differentiated categories (11). HPV status was evaluated via immunohistochemical staining for p16; staining intensity was quantified as 0-+, ++, +++, and++++, with the latter two categories considered positive for p16 (12). mPR was defined as the presence of ≤10% residual viable tumor in the resected tissue, while pCR was indicative of the absence of residual malignant lesions. The combined positive score (CPS) was utilized to appraise the proportion of PD-L1-positive tumor and infiltrating immune cells in relation to the total viable tumor cells (13). Radiological responses were evaluated in adherence with the Response Evaluation Criteria in Solid Tumors version 1.1 (14). Time to surgery (TTS) was defined as the duration between the completion of NAIC and surgery.

Primary endpoints of interest were mPR and pCR. Secondary endpoints included surgical complication and 3-year disease free survival (DFS). Complications were ascertained in accordance with the Clavien-Dindo Classification system, wherein grade I-II incidents were categorized as minor, whereas grade III-V occurrences were considered major (15). The DFS time was computed from the initiation of curative therapy to the onset of recurrence or death, or the final follow-up.

### Therapeutic interventions

The NAIC regimen consisted of docetaxel at a dosage of 75 mg/m², cisplatin at 75 mg/m², and pembrolizumab or alternative PD-1 inhibitors at 200 mg per three-week cycle for two to more cycles. Surgery was determined following multidisciplinary consultation of physical and imaging examination. Resection margins were established based on pre-neoadjuvant therapy evaluations and remained consistent despite therapeutic response. Adjuvant therapy was initiated within six weeks post-surgery, focusing on the tumor bed with a 1-2 cm margin.

### Statistical analysis

Missing data were minimal (<2% for any variable) and handled using complete-case analysis.

The impact of TTS on primary outcome and surgical complication was initially assessed using the Chi-square test, followed by logistic regression analysis, with results expressed as odds ratios (OR) and 95% confidence intervals (CI). The influence of TTS on DFS was evaluated through univariate and Cox regression analyses, with results presented as hazard ratios (HR) and 95% CI. All statistical computations were performed using R version 3.4.4, with a p-value of <0.05 deemed statistically significant.

To minimize the potential impact of confounding factors on the association between TTS and the outcomes of interest, we employed multivariable regression models (logistic regression for pathological responses and complications, Cox regression for DFS). All variables with a p-value < 0.10 in univariate analysis were considered for inclusion in the initial multivariable models. A backward stepwise selection procedure was then applied to retain only variables with p < 0.05 in the final models. This approach aims to adjust for the influence of these covariates on the estimated effect of TTS.

To evaluate the predictive accuracy of the multivariable models, we calculated the c-statistic for logistic regression models and the concordance index (C-index) for Cox proportional hazards models. These measures quantify the model’s ability to discriminate between patients with and without the outcome of interest, with values ranging from 0.5 (chance discrimination) to 1.0 (perfect discrimination).

## Results

### Baseline data

A total of 356 patients, with a median age of 50 years (range: 24–78 years), were enrolled in the study. The cohort comprised 220 males (61.8%) and 136 females (38.2%). The Eastern Cooperative Oncology Group (ECOG) performance score was 0 in 154 patients (43.2%) and 1 in 202 patients (56.8%). A majority of patients were smokers (n=222, 62.4%) and drinkers (n=156, 43.8%). Expression of p16 was positive in 20 patients (5.6%). The most common primary site was the oral cavity (n=200, 56.2%), followed by the hypopharynx (n=56, 15.7%), oropharynx (n=50, 14.0%), and larynx (n=50, 14.0%). Pathological differentiation was well in 110 patients (30.9%), moderate in 139 (39.0%), and poor in 107 (30.1%). According to the AJCC 8th edition staging system, 120 patients (33.7%) presented with clinical stage III disease, 180 (50.6%) with stage IVA, and 56 (15.7%) with stage IVB. Following NAIC, pathological staging revealed ypT0 in 117 patients (32.9%), ypT1/2 in 120 (33.7%), and ypT3/4 in 119 (33.4%); nodal status was ypN0 in 245 patients (68.8%), ypN1/2 in 86 (24.2%), and ypN3 in 25 (7.0%). The majority of patients (65.4%) received two cycles of NAIC, while 78 (21.9%) received three cycles, and 45 (12.6%) received four cycles. Approximately two-thirds of patients underwent surgery within two weeks after NAIC completion, while 61 cases (17.1%) underwent surgery more than three weeks after NAIC. An R0 resection was achieved in 351 patients (98.6%), while five (1.4%) had microscopically positive margins. Postoperatively, 284 patients (79.8%) received adjuvant radiotherapy alone, and 72 (20.2%) received chemoradiotherapy. When analyzed by TTS, the proportion of patients with R0 resection was 99.0% (292/295) in the TTS ≤3 weeks group and 96.7% (59/61) in the TTS >3 weeks group; this difference was not statistically significant (p = 0.184). ENE was identified in 25 patients overall (7.0%). The incidence of ENE was 6.1% (18/295) in the TTS ≤3 weeks cohort compared to 11.5% (7/61) in the TTS >3 weeks cohort, a difference that also did not reach statistical significance (p = 0.140). (**Table 1**).

**Table 1:** Baseline and clinical characteristics of the enrolled patients.

Variable	Number (%)
Age	
≤50	189 (53.1%)
>50	167 (46.9%)
Sex	
Male	220 (61.8%)
Female	136 (38.2%)
ECOG performance score	
0	154 (43.2%)
1	202 (56.8%)
Smoker	222 (62.4%)
Drinker	156 (43.8%)
p16 status	
Negative	336 (94.4%)
Positive	20 (5.6%)
Primary site	
Oral cavity	200 (56.2%)
Oropharynx	50 (14.0%)
Larynx	50 (14.0%)
Hypopharynx	56 (15.7%)
Pathologic differentiation	
Well	110 (30.9%)
Moderate	139 (39.0%)
Poor	107 (30.1%)
Clinical Stage (AJCC 8th ed.)	
III	120 (33.7%)
IVA	180 (50.6%)
IVB	56 (15.7%)
Pathological Stage after NAIC (ypTNM)	
ypT0	117 (32.9%)
ypT1/2	120 (33.7%)
ypT3/4	119 (33.4%)
ypN0	245 (68.8%)
ypN1/2	86 (24.2%)
ypN3	25 (7.0%)
Cycles of NAIC	
Two	233 (65.4%)
Three	78 (21.9%)
Four	45 (12.6%)
Time to Surgery (weeks)	
<1	99 (27.8%)
1–2	121 (34.0%)
2–3	75 (21.1%)
>3	61 (17.1%)
Margin status	
Negative	351 (98.6%)
Positive	5 (1.4%)
Adjuvant Therapy	
Radiotherapy alone	284 (79.8%)
Chemoradiotherapy	72 (20.2%)

### pCR and mPR

**Figure 1:**
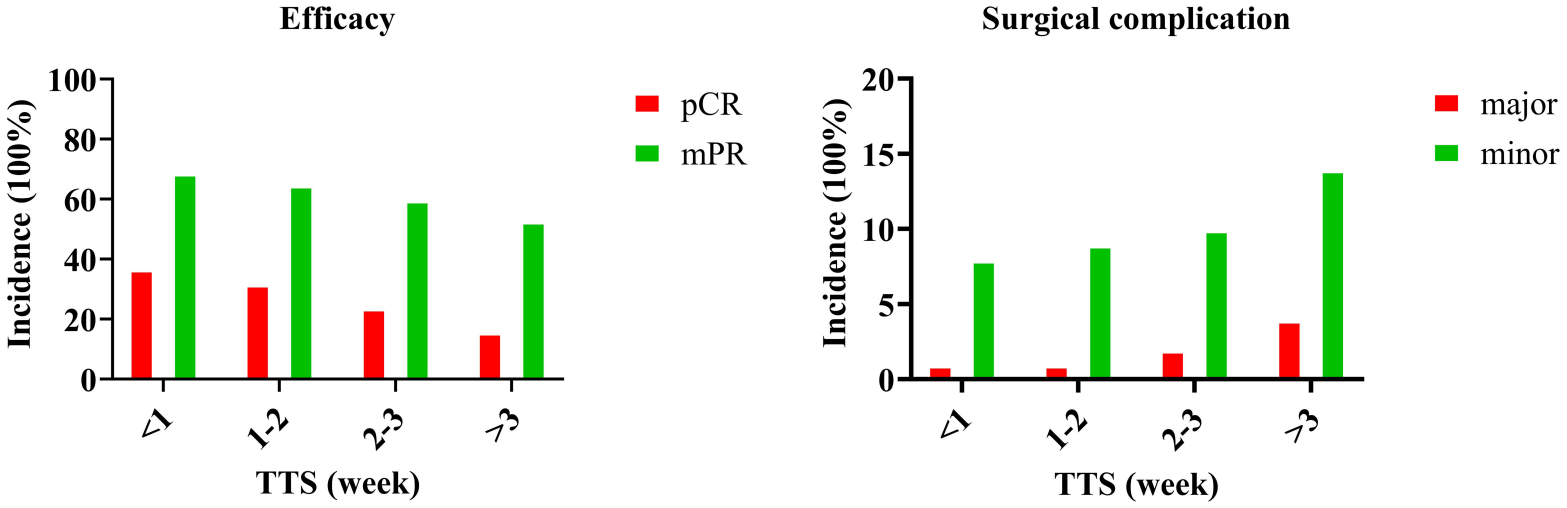
Efficacy and surgical complication in patients with different time to surgery (TTS).

A total of 225 patients (63.2%) achieved a mPR, among whom a pCR was achieved in 104 patients (29.2%). The pCR rates were distributed as follows: 37% in the TTS group of less than 1 week, 32% in the 1-2 week group, 24% in the 2-3 week group, and 16% in the greater than 3 week group, with a significant difference observed between these groups (**Figure 1**). Two additional significant variables were identified as the primary site of the tumor and the degree of pathological differentiation (**Supplementary Table 1**). These three factors were subjected to further analysis using logistic regression. When compared to the TTS<1 week group, patients with a TTS of 1-2 weeks and those with a TTS of 2-3 weeks exhibited comparable rates of pCR achievement; however, patients who underwent surgery more than 3 weeks after the completion of NAIC were significantly less likely to achieve total tumor regression by 16% (95% CI: 1%-23%), with a p-value of 0.043. The multivariable logistic regression model for pCR demonstrated moderate discriminative ability (c-statistic = 0.71, 95% CI: 0.66-0.76) (**Table 2**).

**Table 2:** Independent impact of time to surgery (TTS) on pathologic complete response (pCR), major pathologic response (mPR) and surgical complication.

TTS (week)	pCR		mPR		Complication	
	p	OR [95%CI]	p	OR [95%CI]	p	OR [95%CI]
<1		ref		ref		ref
1-2	0.437	1.12 [0.76-1.78]	0.562	1.23 [0.75-1.80]	0.365	1.24 [0.68-1.87]
2-3	0.205	1.04 [0.59-1.54]	0.205	1.07 [0.62-1.62]	0.087	1.35 [0.90-2.00]
>3	0.043	0.84 [0.77-0.99]	0.031	0.79 [0.64-0.95]	0.021	1.67 [1.11-2.14]

The rates of mPR were 69% in the TTS<1 week group, 65% in the 1-2 week group, 60% in the 2-3 week group, and 53% in the >3 week group, with a significant difference noted between these groups (**Figure 1**). The primary site of the tumor and the degree of pathological differentiation were again identified as significant variables (**Supplementary Table 2**). Logistic regression analysis of these three factors revealed that, when compared to the TTS<1 week group, patients with a TTS of 1-2 weeks and those with a TTS of 2-3 weeks had similar rates of mPR achievement, yet patients who underwent surgery more than 3 weeks after NAIC completion exhibited a significantly lower probability of major tumor regression by 21% (95% CI: 5%-36%), with a p-value of 0.031. The multivariable logistic regression model for mPR demonstrated acceptable discriminative ability (c-statistic = 0.73, 95% CI: 0.68-0.78) (**Table 2**).

### Surgical complication

Postoperative complications comprised surgical site infections (n=34), wound dehiscence (n=6), flap re-exploration (n=4), chylous fistula (n=3), and flap necrosis (n=2). Of these complications, eight cases were classified as major and forty-one as minor. When analyzed separately, neither minor nor major complications showed a statistically significant association with TTS categories in univariate analysis (both p-values > 0.05). Therefore, to increase statistical power, all complications were combined into a composite endpoint (“any complication”) for subsequent multivariable analysis. A high ECOG performance score and a history of smoking were found to be associated with an increased likelihood of surgical complications in univariate analysis (**Supplementary Table 3**). These two factors, along with TTS categories, were included in a multivariable logistic regression model. When compared to the TTS <1 cohort, the TTS ranging from 1 to 2 weeks exhibited no significant impact on the incidence of surgical complications (OR 1.24, p=0.365). Patients in the TTS 2-3 weeks group showed a trend toward higher complication risk (OR 1.35, p=0.087), and those in the TTS >3 weeks group had a significantly increased risk, with an odds ratio of 1.67 (95% CI: 1.11-2.14, p=0.021). The final model for surgical complications showed limited discriminative capacity (c-statistic = 0.65, 95% CI: 0.59-0.71) (**Table 2**).

### DFS

**Figure 2:**
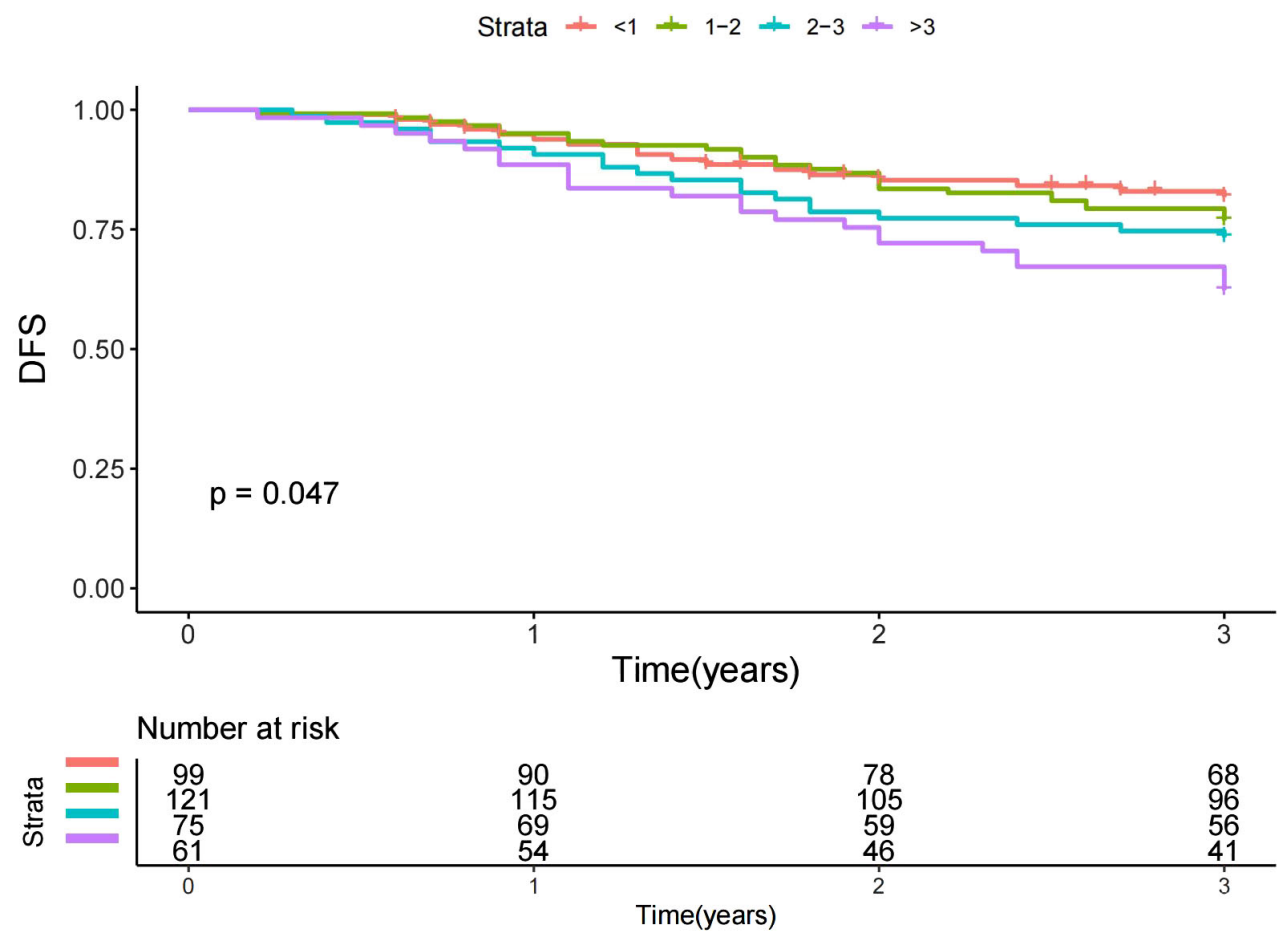
Disease free survival (DFS) in patients with different time to surgery (TTS).

Throughout our median follow-up period of three years, we documented 88 adverse events, which included 18 deaths and 70 instances of recurrence. Univariate analysis revealed that factors such as TTS (**Figure 2**), primary site, pathological response, ypT stage, and ypN stage were correlated with DFS (**Table 3**, all p-values < 0.05). In the subsequent Cox proportional hazards model, when compared to the TTS<1 cohort, intervals of TTS ranging from 1 to 2 weeks and from 2 to 3 weeks did not significantly affect DFS. However, a TTS >3 weeks was found to presage a 1.23-fold increased risk of adverse events (p=0.035). Other independent prognostic factors included the primary site of the tumor, pathological response, ypT stage, and ypN stage. The Cox proportional hazards model for DFS exhibited good discriminative ability (C-index = 0.75, 95% CI: 0.70-0.80) (**Table 3**).

**Table 3:** Univariate and multivariable analysis of predictors for 3-year DFS.

Variable	Univariate	Multivariable	
	Log-rank	p	HR [95%CI]
Age (>50 vs ≤50)	0.432		
Sex (male vs female)	0.731		
ECOG PS (1 vs 0)	0.317		
Smoker	0.813		
Drinker	0.435		
p16 (positive vs negative)	0.589		
Primary site			
Oral cavity			ref
Oropharynx		<0.001	1.78 [1.12-2.34]
Larynx		0.278	1.02 [0.45-1.99]
Hypopharynx	<0.001	<0.001	2.11 [1.24-2.90]
Pathologic response			
pCR			ref
mPR but not pCR		0.114	1.10 [0.32-1.87]
No mPR	<0.001	<0.001	1.79 [1.20-2.63]
Cycle of NAIC (4 vs 3 vs 2)	0.336		
Time to surgery (week)			
<1			ref
1-2		0.453	1.00 [0.43-1.67]
2-3		0.379	1.11 [0.54-1.72]
>3	0.047	0.035	1.23 [1.01-1.68]
Perineural invasion (yes vs no)	0.437		
Lymphovascular invasion (yes vs no)	0.143		
ypT			
ypT0			ref
ypT1/2		0.013	1.34 [1.07-1.70]
ypT3/4	<0.001	<0.001	1.83 [1.14-2.45]
ypN			
ypN0			ref
ypN1/2		0.009	1.20 [1.04-1.76]
ypN3	<0.001	<0.001	1.55 [1.22-1.89]
Adjuvant therapy*			
RT			
CRT	0.479		
Margin status			
Negative			
Positive	0.738		

* RT: radiotherapy; CRT: chemoradiation.

## Discussion

Current study constitutes the inaugural report suggesting that in HNSCC patients following NAIC, on the one hand, a TTS exceeding 3 weeks is independently associated with a diminished likelihood of achieving both pCR and mPR, as well as a truncated duration of DFS. On the other hand, neither p16 status nor the number of treatment cycles impacts the pathological response. Our findings provide important observational data relevant to determining the optimal timing for surgery subsequent to NAIC, indicating that the intervals longer than 3 weeks may be suboptimal. This adds valuable evidence to inform the clinical decision-making process in HNSCC management.

Immunochemotherapy has garnered considerable attention for its promising role in the neoadjuvant setting, a notion validated by multiple clinical trials. Huang et al. (5) prospectively enrolled 20 patients, achieving a remarkable 100% completion rate for neoadjuvant therapy and subsequent R0 resection. The mPR rate was recorded at 60%, inclusive of a 30% pCR. Notably, mPR was attained in all four patients exhibiting a combined positive score of PD-L1 greater than 10. During a median follow-up of 23 months, DFS reached an impressive 90%, while overall survival stood at 95%. In another trial involving 48 patients (8), 27 proceeded to surgical resection, while the remaining 21 received non-surgical therapy. The objective response rate among the 48 patients who completed neoadjuvant therapy was 89.6%. Among the 27 patients who underwent surgical intervention, 17 achieved either mPR or pCR, yielding a pCR rate of 55.6%. These encouraging outcomes align with findings from other reports (13, 16). However, these studies collectively overlooked the potential influence of TTS. Adverse events during NAIC are not infrequent, necessitating judicious pharmaceutical management prior to surgery, and the interim period may also entail the risk of tumor progression. The impact of the interlude between neoadjuvant therapy and surgery on pathological responses is a subject of considerable interest in the prognostication of esophageal cancer survival. However, the inferences drawn from various studies exhibit marked discrepancies. Some investigations have discerned that the temporal gap from the initiation of neoadjuvant radiotherapy and chemotherapy to surgery is not correlated with the incidence of a pCR or overall survival. Studies by Nielsen (17) and Chiu et al. (18) have even suggested that prolonging this interval does not augment the pCR rate and may signify a less favorable survival outlook. Conversely, other reports have indicated that an extended interval, while not enhancing survival rates, is associated with a higher pCR rate and may imply a more favorable prognostic outcome (19, 20). In contradistinction to esophageal cancer, where neoadjuvant chemotherapy alone tends to confer a circumscribed survival advantage in HNSCC, NAIC remains the modality of choice. The quintessential timing for surgical intervention has yet to be comprehensively elucidated. The question of whether a delay in surgery influences the tumor’s pathological response, as well as the outcomes for survival, persists in ambiguity. Moreover, there is a paucity of research that delineates this issue with clarity.

In the present investigation, we observed a significant decrement in both the rates of pCR and mPR within the cohort characterized by a TTS exceeding three weeks. Additionally, a poorer DFS was documented in this TTS >3 group. This finding is of paramount importance, suggesting that surgery should ideally be performed within no more than three weeks following NAIC. Regrettably, there exists a scarcity of related literature for comparative analysis, although this subject has been explored within the context of esophageal squamous cell carcinoma. Yang et al. (10) stratified 152 patients into short-interval (time to surgery ≤ 6 weeks) and long-interval groups (time to surgery > 6 weeks), with pCR rates of 34.1% and 24.6% respectively. Delayed surgery did not significantly affect the number of lymph node dissections, operative duration, blood loss, hospitalization period, chest drainage duration, or postoperative complications. The three-year overall survival rates were 85.10% in the short-interval group and 82.07% in the long-interval group, with corresponding three-year DFS rates of 83.41% and 70.86%. Another study involving 80 patients categorized them based on the interval from neoadjuvant immunochemotherapy to surgery: ≤ 8 weeks (n = 44) and > 8 weeks (n = 36) (21). The mPR rates were 25.0% in the ≤ 8 weeks group and 27.8% in the > 8 weeks group, while pCR rates were 11.4% and 16.7% respectively. Multivariable analysis revealed inferior DFS and overall survival in patients with an interval time exceeding 8 weeks. A recent study (9) demonstrated pCR rates of 23.4% in the timely surgery group (<6 weeks) and 12.8% in the delayed surgery group (>6 weeks). The respective three-year overall survival rates were 77.5% and 63.5%, with DFS rates of 59.1% and 38.4%. Multivariate Cox regression analyses indicated that the interval from immunochemotherapy to surgery was an independent prognostic factor for both overall survival and DFS. These studies present a spectrum of inconsistent results, which are partly in conflict with and partly in concordance with our analysis. Initially, delayed surgery due to poor physical condition is a pivotal factor impacting survival outcomes. Secondly, a protracted interval following neoadjuvant therapy may be a risk factor for metastasis, and such intervals are more likely to occur in patients with advanced clinical lymph node staging and larger tumor lesions. Lastly, a 3-week interval aligns with one cycle of NAIC, and in reality, three or more cycles do not confer higher efficacy or improved DFS, merely affording additional time for tumor progression.

In current study, the preponderance of complications were of a minor nature, obviating the need for surgical intervention. Despite the absence of a comparative analysis, we nonetheless maintain the belief that NAIC did not augment the incidence of complications, a perspective that has been previously validated (22). It is intriguing to observe that a prolonged TTS correlates with a higher complication rate. One possible explanation for this association could be that the need for a longer interval may reflect a poorer recovery from NAIC or underlying patient frailty. This observation highlights the importance of careful patient assessment and potentially more frequent monitoring when surgery is delayed. This subject has been previously analyzed in the context of other solid tumors. Liu et al. (21) further suggest that there is no significant discrepancy in the incidence of postoperative complications between the delayed surgery group and the timely surgery group in cases of esophageal squamous cell carcinoma. However, Overtoom et al. (23) discovered that surgery subsequent to neoadjuvant chemoradiotherapy heightens the risk of postoperative respiratory system afflictions in esophageal cancer patients. Wang and colleagues (24) also observed that an extended interval between neoadjuvant chemotherapy and surgery is positively correlated with an elevated risk of exercise-induced fistula recurrence. This matter necessitates additional clinical trials for comprehensive investigation.

In clinical practice, the number of cycles of neoadjuvant immunochemotherapy administered to most patients with HNSCC is typically determined to be two or three based on anecdotal experience rather than established guidelines. Notably, no comparative studies have evaluated the efficacy of two versus other cycles specifically in HNSCC. However, this scientific inquiry has garnered substantial attention in the context of other solid malignancies. A recent investigation (25) involving 108 patients with non-small cell lung carcinoma revealed that patients in the two-cycle group exhibited significantly smaller diagnostic radiological tumor size (37.0 mm versus 49.6 mm, p = 0.022) and a higher radiological tumor regression rate (36% versus 49%, p = 0.007) compared to those receiving more than two cycles. Nevertheless, no statistically significant difference in pathological tumor regression rate was observed between the two-cycle group and those undergoing more than two cycles. Conversely, additional evidence suggests a negative correlation regarding the number of cycles. In a study encompassing 176 patients with non-small cell carcinoma (26), the ORRs for those receiving two, three, four, and five or more cycles were recorded at 52%, 67%, 53%, and 50%, respectively. *Post hoc* analysis indicated that the number of treatment cycles bore no significant association with mPR or pCR. Furthermore, treatment cycles did not impact operational performance metrics, such as operating time, postoperative drainage, and length of hospital stay. A meta-analysis (27) elucidated that neoadjuvant immunochemotherapy correlates with improved 2-year DFS and elevated pCR rates in experimental versus control treatment arms, with this association remaining unaffected by tumor- or treatment-related factors, including PD-L1 status, the type of platinum-based chemotherapy, or the number of cycles in non-small cell carcinoma. Our study aligns with this perspective, suggesting that the number of neoadjuvant therapy cycles does not significantly influence any outcome variables. Consequently, our findings provide initial evidence that two cycles may represent a rational and effective choice in the context of HNSCC.

The limitations of the current study must be acknowledged. Firstly, the retrospective design introduces an inherent risk of selection bias. The timing of surgery was determined by clinical judgment rather than randomization, potentially influenced by unrecorded factors such as the severity of treatment-related adverse events, patient comorbidities, or logistical delays. Although we adjusted for available clinicopathological variables in our multivariable analyses and found no significant differences in major baseline and treatment characteristics between TTS groups, residual confounding from unmeasured or imperfectly measured variables may persist. Secondly, our sample size, particularly within the TTS >3 weeks subgroup (n=61), is relatively modest, which may limit the statistical power for some comparisons and the generalizability of the findings. Lastly, this investigation was conducted at a single institution. The patient population, NAIC protocols, and surgical practices may differ from other centers, necessitating external validation in multi-institutional cohorts or prospective settings before definitive clinical recommendations can be established.

In conclusion, a TTS exceeding 3 weeks was independently associated with a lower likelihood of achieving pCR and mPR, as well as a shorter DFS. Our data support the hypothesis that delaying surgery beyond 3 weeks after NAIC completion may be detrimental, and suggest that aiming for a surgical interval within 3 weeks could be beneficial for patients with HNSCC.

## Data Availability

The original contributions presented in the study are included in the article/**Supplementary Material**. Further inquiries can be directed to the corresponding author.
