# Supplementary material for: Optimal timing of surgery in head and neck squamous cell carcinoma after neoadjuvant immunochemotherapy
Source: Front Oncol. 2026 Feb 6;16:1742883. doi: 10.3389/fonc.2026.1742883 (PMC12920233; doi:10.3389/fonc.2026.1742883)
Supplement: Supplementary file 1 [file Table1.doc]

Supplementary Table 1. Predictors for pathologic complete response.

| Variable | Univariate | Logistic regression | |
| --- | --- | --- | --- |
|  | p | p | OR [95%CI] |
| Age |  |  |  |
| ≤50 |  |  |  |
| >50 | 0.537 |  |  |
| Sex |  |  |  |
| Male |  |  |  |
| Female | 0.376 |  |  |
| ECOG performance score |  |  |  |
| 0 |  |  |  |
| 1 | 0.704 |  |  |
| Smoker | 0.280 |  |  |
| Drinker | 0.325 |  |  |
| p16 |  |  |  |
| Negative |  |  |  |
| Positive | 0.107 |  |  |
| Primary site |  |  |  |
| Oral cavity |  |  | ref |
| Oropharynx |  | 0.008 | 1.45 [1.13-1.87] |
| Larynx |  | 0.114 | 1.20 [0.56-1.66] |
| Hypopharynx | <0.001 | 0.005 | 1.51 [1.15-1.90] |
| Pathologic differentiation |  |  |  |
| Well |  |  | ref |
| Moderate |  | 0.134 | 1.11 [0.78-1.48] |
| Poor | <0.001 | 0.003 | 1.34 [1.06-1.76] |
| Cycle of neoadjuvant therapy |  |  |  |
| Two |  |  |  |
| Three |  |  |  |
| Four | 0.335 |  |  |
