# Supplementary material for: Optimal timing of surgery in head and neck squamous cell carcinoma after neoadjuvant immunochemotherapy
Source: Front Oncol. 2026 Feb 6;16:1742883. doi: 10.3389/fonc.2026.1742883 (PMC12920233; doi:10.3389/fonc.2026.1742883)
Supplement: Supplementary file 2 [file Table2.doc]

Supplementary Table 2. Predictors for major pathologic response.

| Variable | Univariate | Logistic regression | |
| --- | --- | --- | --- |
|  | p | p | OR [95%CI] |
| Age |  |  |  |
| ≤50 |  |  |  |
| >50 | 0.426 |  |  |
| Sex |  |  |  |
| Male |  |  |  |
| Female | 0.283 |  |  |
| ECOG performance score |  |  |  |
| 0 |  |  |  |
| 1 | 0.612 |  |  |
| Smoker | 0.315 |  |  |
| Drinker | 0.443 |  |  |
| p16 |  |  |  |
| Negative |  |  |  |
| Positive | 0.328 |  |  |
| Primary site |  |  |  |
| Oral cavity |  |  | ref |
| Oropharynx |  | 0.005 | 1.41 [1.11-1.92] |
| Larynx |  | 0.123 | 1.14 [0.60-1.72] |
| Hypopharynx | <0.001 | 0.001 | 1.52 [1.17-2.00] |
| Pathologic differentiation |  |  |  |
| Well |  |  | ref |
| Moderate |  | 0.153 | 1.12 [0.64-1.50] |
| Poor | <0.001 | 0.001 | 1.35 [1.11-1.68] |
| Cycle of neoadjuvant therapy |  |  |  |
| Two |  |  |  |
| Three |  |  |  |
| Four | 0.287 |  |  |
