# Supplementary material for: Optimal timing of surgery in head and neck squamous cell carcinoma after neoadjuvant immunochemotherapy
Source: Front Oncol. 2026 Feb 6;16:1742883. doi: 10.3389/fonc.2026.1742883 (PMC12920233; doi:10.3389/fonc.2026.1742883)
Supplement: Supplementary file 3 [file Table3.doc]

Supplementary Table 3. Predictors for surgical complication.

| Variable | Univariate | Logistic regression | |
| --- | --- | --- | --- |
|  | p | p | OR [95%CI] |
| Age |  |  |  |
| ≤50 |  |  |  |
| >50 | 0.125 |  |  |
| Sex |  |  |  |
| Male |  |  |  |
| Female | 0.436 |  |  |
| ECOG performance score |  |  |  |
| 0 |  |  | ref |
| 1 | 0.034 | 0.100 | 1.23 [0.83-1.87] |
| Smoker | 0.035 | 0.015 | 1.17 [1.08-1.69] |
| Drinker | 0.243 |  |  |
| p16 |  |  |  |
| Negative |  |  |  |
| Positive | 0.438 |  |  |
| Primary site |  |  |  |
| Oral cavity |  |  |  |
| Oropharynx |  |  |  |
| Larynx |  |  |  |
| Hypopharynx | 0.331 |  |  |
| Pathologic differentiation |  |  |  |
| Well |  |  |  |
| Moderate |  |  |  |
| Poor | 0.745 |  |  |
| Cycle of neoadjuvant therapy |  |  |  |
| Two |  |  |  |
| Three |  |  |  |
| Four | 0.156 |  |  |
